# Bowtie Technique for Arthroscopic Hip Inversion Labral Repair

**DOI:** 10.1002/atn2.70031

**Published:** 2026-04-29

**Authors:** Mathew Park, Spencer Sims, Tyler Bilden, Grace Peterson, Travis Menge

**Affiliations:** ^1^ Corewell Health West Department of Orthopaedic Surgery Grand Rapids Michigan U.S.A.; ^2^ Michigan State University College of Human Medicine Grand Rapids Michigan U.S.A.

## Abstract

Various suture techniques for arthroscopic hip labral repair have been described to address labral pathology. However, optimal restoration of the labral suction seal relies on proper tension and control of the labral tissue during the repair. To enhance the reproducibility and efficiency of labral repair, we describe the Bowtie suture technique that generates an inversion force vector within the repair construct. This suture passing technique reduces the technical complexity of inversion labral repairs by making it significantly easier to pass and retrieve the repair suture. Folding the suture twice and pinching it in the center of the fold forms a Bowtie‐like shape. Then, this Bowtie suture configuration is inserted through a cannula using a suture passing device and placed into the central compartment. The Bowtie is released, allowing it to expand like a spring and hold its position, preventing it from falling into the inferior joint. The suture passer is then placed through the chondrolabral junction, and the repair stitch is easily retrieved and secured to an anchor. This technique improves suture management and efficiency while also allowing for improved control of the labrum to compress the chondrolabral junction and restore the suction seal mechanism.

## Graphical abstract

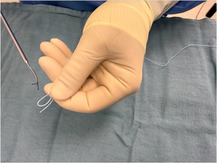

**VIDEO 1:** In typical inversion labral repairs, when the repair suture is released into the joint space without any modifications, technical difficulties can be experienced as the repair suture falls into the inferior joint space. The Bowtie suture technique helps eliminate this technical difficulty often experienced in inversion labral repairs. To create the Bowtie configuration, the repair suture is first folded twice and then pinched in the middle with the jaws of an SwiftStitch suture passer (Arthrex). Viewing from the anterolateral portal, the SwitchStitch suture passer (Arthrex) is advanced through the instrument cannula (Arthrex) into the joint space with the Bowtie suture still intact. The repair suture is then released, and it expands like a spring within the joint space. This expansion of the suture keeps it in place, preventing it from falling into the inferior joint space. The SwiftStitch suture passer (Arthrex) is then passed through the chondrolabral junction for easier retrieval of the repair suture. Video content can be viewed at https://doi.org/10.1002/atn2.70031.

The acetabular labrum is a fibrocartilaginous ring that encircles the rim of the acetabulum and deepens the hip socket. The labrum enhances joint congruity, distributes load, and preserves the negative pressure “suction seal” that supports normal biomechanics and protects articular cartilage.^1^, ^2^, ^3^ When the labrum is torn, this suction seal can be damaged, which compromises the stability of the hip joint.^2^, ^3^ Hip instability symptoms manifest with groin pain, decreased range of motion, and functional limitations.^4^, ^5^

The objective of a labral repair is to restore the negative pressure suction seal between the femoral head and acetabular rim that has been compromised.^2^, ^3^, ^6^ There is debate on which repair techniques allow for optimal refixation position and anatomic restoration of this suction seal, especially for inversion labral repairs.^1^, ^6^ We describe an arthroscopic suture repair technique that not only accomplishes improved restoration of the suction seal, but also provides compression of the labral tearing at the chondrolabral junction to enhance healing potential. While often viewed as technically more challenging, an inversion approach is preferred by the senior author when compared with eversion methods due to the inversion technique's ability to better restore the native suction seal between the labrum and femoral head. The complexity of an inversion labral repair is mitigated with the Bowtie technique, which allows for easier retrieval of the passing suture, improves suture management efficiency, and maintains excellent suture control and fixation of the labrum to the acetabular rim.

## SURGICAL TECHNIQUE

An inversion labral repair using the traditional repair suture configuration as well as demonstrating the Bowtie suture technique is shown in Video 1. Advantages and pitfalls of this technique are described in Table 1.

**TABLE 1:** Advantages and Pitfalls of the Bowtie Suture Technique for Hip Labral Repair

Advantages
• Improved suture management with more efficient suture passing and retrieval • Inversion technique compresses chondrolabral junction to enhance healing • Restores suction seal with femoral head • Reproducible tensioning of labral repair • Avoids labral eversion • Improved OR efficiency

Pitfalls
• Technically more challenging repair technique • Requires detailed suture management to avoid entanglement • Learning curve required

OR, operating room.

### Patient Positioning and Diagnostic Arthroscopy

The patient is placed in a modified supine position, starting with the hip in flexion and neutral rotation. The patient is placed in slight Trendelenburg position, usually about 5° to 10°, which significantly reduces the pressure of the post on the perineum. The hip is then slowly extended from a flexed position while applying abduction and mild internal rotation to help open the joint space, as shown by the vacuum sign, which can be confirmed with radiograph. The 2 primary portals used are the standard anterolateral and the mid‐anterior portal. If a specific case requires an accessory portal, a distal accessory anterolateral portal can be placed. A small interportal capsulotomy is made while making sure to leave at least 5 mm or more of the proximal cuff of capsular tissue off the acetabulum, ensuring sufficient tissue to facilitate adequate closure of the capsule and preservation of the native joint's biomechanics at the end of the procedure.^1^, ^7^, ^8^, ^9^, ^10^ A standard diagnostic hip arthroscopy is then performed.

### Labral Preparation

After diagnostic arthroscopy is complete, a probe is inserted into the chondrolabral junction to confirm the labral tear and assess for labral destabilization.^5^, ^6^, ^7^ The acetabular rim underlying the area of labral tearing is prepared using an 4.5 mm Round Burr (Arthrex, Naples, FL) in preparation for anchor placement.^1^, ^3^, ^4^, ^6^, ^7^, ^9^

### Inversion Labral Repair Using the Bowtie Suture Technique

**FIGURE 1:**
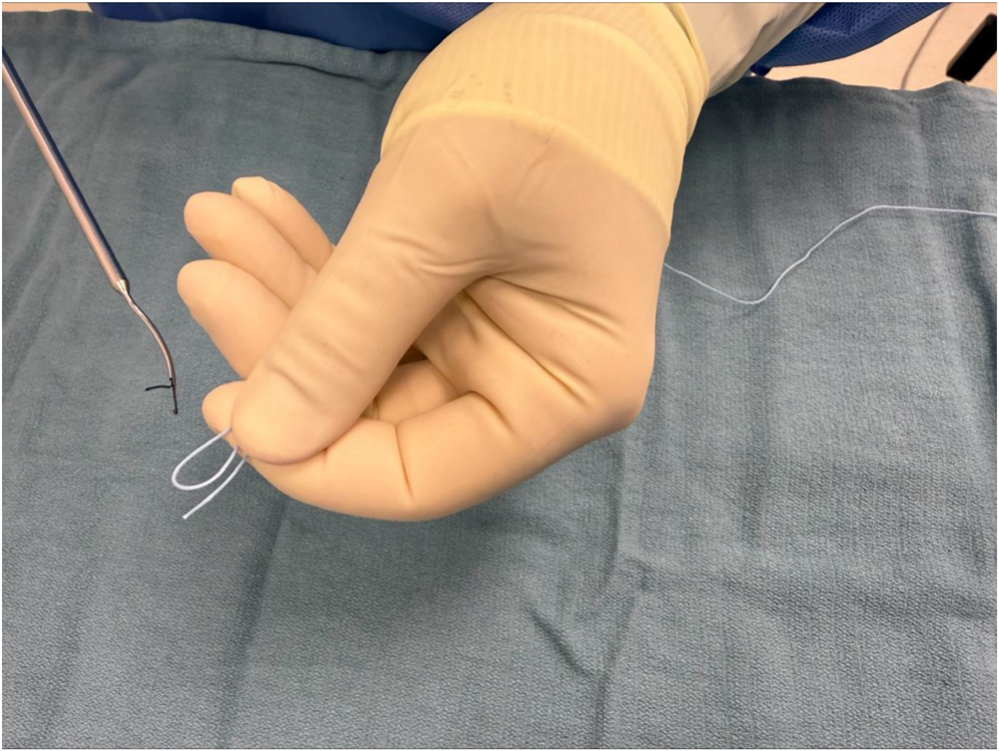
Repair suture folded twice. Image of the hip labral repair suture being prepped to form the Bowtie structure. The repair suture must be folded twice before grasping it in the middle with the SwiftStitch suture passer (Arthrex) to properly form the Bowtie.

**FIGURE 2:**
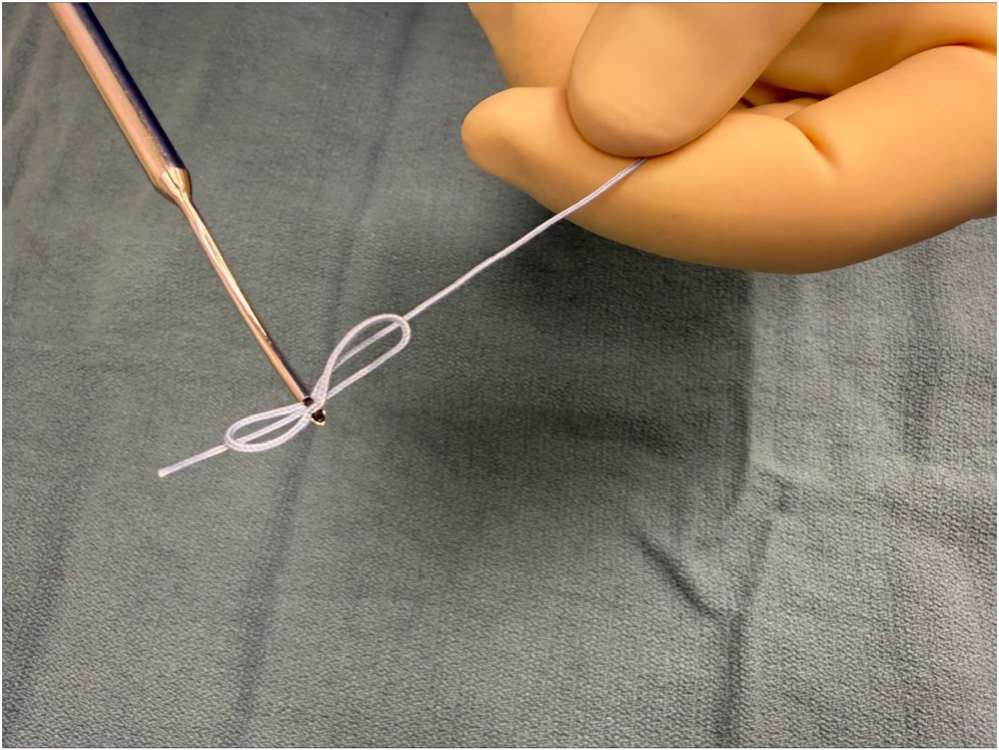
Bowtie suture construct. Image of the Bowtie repair suture prior to being inserted into the arthroscopic instrument cannula (Arthrex) for the inversion labral repair. After folding the repair suture twice, a SwiftStitch suture passer (Arthrex) is used to pinch the suture in the middle of the folds. The Bowtie suture is then complete and ready to be inserted into the hip joint.

A Curved Hip FiberTak, 16° Crown Tip Drill Guide (Arthrex) is inserted through an instrument cannula (Arthrex) to the acetabular rim to drill a hole for insertion of the 1.8 mm Knotless Hip FiberTak Soft Anchor (Arthrex).^2^, ^3^, ^4^, ^5^, ^6^, ^7^, ^8^, ^9^, ^10^, ^11^ The handle and Curved Hip FiberTak, 16° Crown Tip Drill Guide (Arthrex) are then removed from the instrument cannula (Arthrex), and the sutures are pulled to set the 1.8 mm Knotless Hip FiberTak Soft Anchor (Arthrex).^2^, ^3^, ^5^, ^7^, ^9^, ^10^, ^11^ Now, the repair suture is folded twice (Figure 1) and pinched in the middle with the jaws of the SwiftStitch Suture Passer (Arthrex) (Figure 2), forming our Bowtie suture construct. With the Bowtie still intact as seen at timestamp 00:53 in Video 1, the SwiftStitch Suture Passer (Arthrex) is then advanced through the instrument cannula (Arthrex), and the suture is pushed into the joint space and released. This allows the Bowtie to expand like a spring, holding the suture in place and preventing it from falling into the inferior joint space. The SwiftStitch Suture Passer (Arthrex) is then passed through the chondrolabral junction, as shown in Video 1 at timestamp 01:15, to easily retrieve the suture. Outside of the instrument cannula (Arthrex), the repair suture is passed through the looped end of the shuttle suture, and the nonlooped end is pulled to shuttle the repair suture through the knotless mechanism of the 1.8 mm Knotless Hip FiberTak Soft Anchor and to set the appropriate tension. The repair suture is then loaded into the suture cutter for final tensioning and cutting. Subsequent 1.8 mm Knotless Hip FiberTak Soft Anchor (Arthrex) are added in the same fashion to complete the final repair construct.

### Postoperative Rehabilitation

Patients undergoing arthroscopic labral repair are often discharged the day of surgery.^4^ Postoperative care includes partial, foot flat weightbearing with crutches for 3 weeks, early physical therapy, and oral analgesics. Physical therapy will include passive hip motion exercises and muscle activation on postoperative day 1.^2^, ^4^, ^7^ At 3 weeks postoperatively, patients begin light strengthening exercises of the abdominal and hip musculature with physical therapy.^1^, ^7^ Full postoperative recovery and return to sports/activity without limitations often ranges from 4 to 6 months.^4^, ^7^

## DISCUSSION

A wide range of suture configurations have been developed for arthroscopic hip labral repair, each with distinct biomechanical and clinical advantages. For example, the simple loop (circumferential) techniques, while commonly used, has been shown to evert the labrum and compromise the restoration of the suction seal, thereby reducing cartilage preservation that is critical for joint stability.^3^, ^6^, ^10^ In a cadaveric study, Nepple et al. showed that through labral (pierced or labral base) repairs, including vertical mattress configurations, they were able to restore pressurization of the hip joint to 92% of native levels, far superior to the 72% reported with simple looped repairs.^10^, ^12^ These findings suggest that suture orientation and placement have a direct impact on labral function. However, while these techniques achieve excellent mechanical outcomes, they often involve complex suture management, increased operative time, and a higher risk of suture pull‐through with further labral tissue damage. In contrast, the Bowtie suture technique is an approach that combines the biomechanical strengths of through labral repairs with improved suture handling. By folding the suture twice and pinching it at the midpoint to create a Bowtie‐shaped loop; this method allows for significantly easier retrieval of the repair suture through the labrum that has been released into the joint space, thereby reducing the complexity of the inversion labral repair. Unlike simple looped sutures that risk eversion, or mattress configurations that can be difficult to retrieve and pull through the tissue, the Bowtie technique enhances suture management, improves operating room efficiency, facilitates precise control of the labral tissue, and enhances reproducibility of restoring the labral suction seal.

## DISCLOSURES

The authors (M.P., S.S., T.B., G.P., T.M.) declare that they have no known competing financial interests or personal relationships that could have appeared to influence the work reported in this paper.
